# Secondary mechanisms of diversification in the human antibody repertoire

**DOI:** 10.3389/fimmu.2013.00042

**Published:** 2013-03-11

**Authors:** Bryan S. Briney, James E. Crowe Jr.

**Affiliations:** ^1^Department of Pathology, Microbiology and Immunology, Vanderbilt University Medical CenterNashville, TN, USA; ^2^Department of Pediatrics, Vanderbilt University Medical CenterNashville, TN, USA; ^3^The Vanderbilt Vaccine Center, Vanderbilt University Medical CenterNashville, TN, USA

**Keywords:** VDJ rearrangement, VH replacement, B Cell Biology, human, insertion/deletion

## Abstract

V(D)J recombination and somatic hypermutation (SHM) are the primary mechanisms for diversification of the human antibody repertoire. These mechanisms allow for rapid humoral immune responses to a wide range of pathogenic challenges. V(D)J recombination efficiently generate a virtually limitless diversity through random recombination of variable (V), diversity (D), and joining (J) genes with diverse non-templated junctions between the selected gene segments. Following antigen stimulation, affinity maturation by SHM produces antibodies with refined specificity mediated by mutations typically focused in complementarity determining regions (CDRs), which form the bulk of the antigen recognition site. While V(D)J recombination and SHM are responsible for much of the diversity of the antibody repertoire, there are several secondary mechanisms that, while less frequent, make substantial contributions to antibody diversity including V(DD)J recombination (or D–D fusion), SHM-associated insertions and deletions, and affinity maturation and antigen contact by non-CDR regions of the antibody. In addition to enhanced diversity, these mechanisms allow the production of antibodies that are critical to response to a variety of viral and bacterial pathogens but that would be difficult to generate using only the primary mechanisms of diversification.

## INTRODUCTION

A diverse antibody repertoire is a principal component of humoral immunity and is critical to the development of functional adaptive immune responses. Generation of this repertoire diversity is accomplished primarily through two mechanisms: recombination and somatic hypermutation (SHM). These two mechanisms produce massive diversity within antibody complementarity determining regions (CDRs), which form the primary antigen contact site. The availability of multiple variable genes for selection at the time of recombination facilitates large combinatorial diversity, which is further expanded by a diversity of possible heavy and light chain combinations. In this review, we discuss in detail three additional mechanisms which, while less common than recombination and SHM, contribute substantially to the generation of diversity within the antibody repertoire: (1) non-standard recombinations that violate the 12/23 rule of recombination, (2) SHM-associated genetic insertions and deletions, and (3) affinity maturation and direct antigen contact by non-CDR antibody regions.

## V(D)J RECOMBINATION: FOLLOWING THE 12/23 RULE

Since the discovery that recombination activating gene (RAG)-mediated recombination of variable (V), diversity (D) and joining (J) genes generates virtually unlimited sequence diversity in the antibody repertoire (Brack et al., 1978; Alt and Baltimore, 1982; Tonegawa, 1983; Schatz et al., 1989; Oettinger et al., 1990), much progress has been made in determining the genetic and mechanistic elements that participate in the antibody recombination process. It is generally understood that recombination signal sequences (RSS), which are composed of conserved AT-rich heptamer and nonamer sequences separated by spacers of either 12 or 23 nucleotides, are recognized and bound by RAG1 and RAG2 proteins at the initiation of the recombination process (Hesse et al., 1989; Alt et al., 1992). RAG binding is highly dependent on the heptamer and nonamer sequences, and alterations to either sequence results in decreased RAG binding (Cuomo et al., 1996; Difilippantonio et al., 1996; Nadel et al., 1998). The length of the spacer sequence is critical to recombination, and there is evidence of sequence conservation within the spacer region (Ramsden et al., 1994; Lee et al., 2003; Montalbano et al., 2003).

Recombination typically occurs only between RSS elements of different spacer lengths, in a model commonly referred to as the 12/23 rule of recombination (Ramsden et al., 1996; Steen et al., 1996; van Gent et al., 1996; Schatz, 2004). After binding to one 12-bp RSS and one 23-bp RSS, the RAG complex induces single-strand DNA nicks between the coding sequence and the heptamer of each RSS, resulting in hairpin formation on each of the coding ends and a blunt double-stranded break on each signal end (Roth et al., 1992; Schlissel et al., 1993; McBlane et al., 1995; Sadofsky, 2001). The hairpins are opened, nucleotides may be added to or removed from the coding ends, and the double-strand DNA breaks at the coding ends are joined into a single coding strand (Lewis, 1994; Mahajan et al., 1999; Shockett and Schatz, 1999; Walker et al., 2001; Mansilla-Soto and Cortes, 2003; Roth, 2003).

In antibody heavy chain genes, D gene segments are flanked by 12-bp RSSs on either side, while V_H_ and J_H_ gene segments are flanked by 23-bp RSSs (Early et al., 1980; Kurosawa and Tonegawa, 1982). Recombination thus proceeds in a step-wise fashion, with D–J_H_ recombination preceding V_H_–D recombination, resulting in a complete heavy chain variable region (Alt et al., 1987; Schatz et al., 1992). A single recombination event joins the light chain V and J gene, and pairing of recombined heavy chain and recombined light chains results in massive diversity within the unmutated antibody repertoire.

## NON-12/23 RECOMBINATION: V(DD)J AND DIRECT V_H_–J_H_ RECOMBINATION

Direct V_H_–J_H_ joining and V(DD)J recombination (also referred to as D–D fusion) are in direct violation of the 12/23 rule, but such recombination events have been demonstrated in both *in vitro* and *in vivo* systems (Sanz, 1991; Kiyoi et al., 1992; Raaphorst et al., 1997; Koralov et al., 2005, 2006; Watson et al., 2006). Even in model systems designed to induce such recombination events, however, non-12/23 recombinations are much less efficient than recombinations that adhere to the 12/23 rule (Akira et al., 1987; Hesse et al., 1989; Akamatsu et al., 1994).

V(DD)J recombinants are the result of an aberrant recombination process by which two or more D genes are joined into a single recombinant. The joining of two D genes, which are flanked on both sides by 12-bp RSSs, can only be accomplished in clear violation of the 12/23 rule, but recombined antibody genes in this configuration have now been isolated by numerous investigators. While V(DD)J recombination typically results in an unusually long heavy chain CDR 3 (HCDR3) region, the use of two D segments is not the primary mechanism by which long HCDR3 loops are generated (Briney et al., 2012a). Long HCDR3s typically are generated by the use of longer D and J segments and long non-templated junctional regions. The precise order of events during the V(DD)J recombination process is unclear: it is not known whether V(DD)J recombinants are produced through an additional D–D recombination following the initial D–J_H_ recombination, or whether D–D fusion occurs before, even long before, the D–J_H_ recombination. V(DD)J recombinations have been estimated by some to occur in as many as 5–11% of all recombinations (Sanz, 1991; Kiyoi et al., 1992; Raaphorst et al., 1997), but the true frequency of V(DD)J recombinations is difficult to determine. Identification of V(DD)J recombinants relies on the accurate detection of two diversity genes within a single recombinant, but N-addition mimicry of diversity gene segments, which is genetically indistinguishable from true V(DD)J recombination, likely inflates many published estimates of V(DD)J recombination (Watson et al., 2006). Recent work, which leveraged high-throughput sequencing and a stringent filtering process, placed a lower bound of the frequency of V(DD)J recombinants in the human peripheral blood repertoire at approximately 1 in 800 B cells (Briney et al., 2012b).

The occurrence of direct V_H_–J_H_ recombination, like V(DD)J recombination, requires clear violation of the 12/23 rule, since both V_H_ and J_H_ segments are flanked by 23-bp RSSs. Little is known about the frequency of direct V_H_–J_H_ recombination in the human repertoire. Several studies of the human CDR3 repertoire that have identified D–D fusions have failed to identify V_H_–J_H_ recombinants, indicating that if they occur, V_H_–J_H_ recombinations are likely very rare (Sanz, 1991; Kiyoi et al., 1992; Raaphorst et al., 1997; Watson et al., 2006). This finding is somewhat surprising, since *in vitro* recombination between two 23-bp RSSs occurred much more frequently than recombination between two 12-bp RSSs (Jones and Gellert, 2002). In contrast to D–D fusions, for which there are several studies on the frequency of V(DD)J recombinants in the human peripheral blood repertoire, much of the published work describing *in vivo* V_H_–J_H_ recombination relies on transgenic mouse models lacking D gene loci (Koralov et al., 2005, 2006). Since these model systems produce only aberrant recombinants, it is difficult to interpret the resulting data in terms of the likely occurrence and frequency of such recombinants in the naturally occurring circulating B cell repertoire. As with V(DD)J recombination, determination of the true frequency of direct V_H_–J_H_ recombination will likely prove difficult, as extensive chewback of D genes during normal V(D)J recombination may appear genetically indistinguishable from true V_H_–J_H_ recombination and inflate any estimates of the frequency of V_H_–J_H_ recombination.

## NON-12/23 RECOMBINATION: V_H_ REPLACEMENT AND RECEPTOR REVISION

V_H_ replacement is a process by which a secondary V_H_–V(D)J recombination can occur, resulting in replacement of the variable gene while preserving the original D–J_H_ recombination. V_H_ replacement, which is though to be a form of heavy chain receptor editing, differs from light chain receptor editing, although both typically occur early in B cell development (Prak and Weigert, 1995; Nemazee and Weigert, 2000). Light chain receptor editing results in an entirely new V_L_–J_L_ recombination through the recombination of a V_L_ gene segment upstream of the original recombination with a J_L_ gene segment downstream of the original recombination (Papavasiliou et al., 1997; Retter and Nemazee, 1998). Thus, light chain receptor editing proceeds without violating the 12/23 rule. In contrast, V_H_ replacement involves V_H_–V(D)J recombination, which results in retention of the original D–J_H_ junction and replacement only of the V_H_ gene segment (Kleinfield and Weigert, 1989; Nemazee, 2006). V_H_ replacement utilizes a cryptic RSS (cRSS) found near the 3′ end of most human variable genes (Radic and Zouali, 1996), and this cRSS is used to recombine with the normal RSS at the 3′ end of the invading variable gene. The cRSS contains a heptamer sequence, but lacks an identifiable nonamer or spacer sequence, and recombination with the cRSS is inefficient, much like other forms of non-12/23 recombination (Koralov et al., 2006; Lutz et al., 2006).

V_H_ replacement also can be distinguished from receptor revision, which is putatively antigen-driven and has not been shown to use the conserved cRSS elements near the 3′ end of the V gene. Instead, receptor revisions are suggested to occur peripherally in mature B cells using alternate RSS-like elements that sometimes contain only the CAC motif found at the 5′ end of most RSS heptamers or the inverse GTG motif found at the 3′ end; the few examples of this phenomenon typically occurred near the middle of heavy chain framework region (FR) 3 (Itoh et al., 2000; Wilson et al., 2000; Lenze et al., 2003). Use of these alternate RSS-like elements results in formation of a hybrid V gene, retaining a substantial portion of the initially recombined V gene, as opposed to the nearly complete removal of the initially recombined V gene observed in V_H_ replacement. Because the observed receptor revision events occurred in stretches of sequence similarity between V genes, it has been proposed that these revisions may instead be polymerase chain reaction (PCR) artifacts caused by incomplete recombinant amplification followed by priming of a different V(D)J recombinant with the partially amplified fragment, resulting in a hybrid sequence (Darlow and Stott, 2005). In approximately half of all identified receptor revisions in these studies, the invading V gene is located downstream of the variable gene used in the initial V(D)J recombination, which would not be possible using the proposed receptor revision mechanism. Inter-chromosomal recombination has been proposed as the mechanism for these out-of-order receptor revisions (Wilson et al., 2000). More recent work has shown that receptor reversions are not observed when amplifying from single B cells (Goossens et al., 2001), providing further evidence that the previously observed receptor revisions may be an artifact of PCR amplification of multiple antibody sequences from bulk B cells.

It is thought that V_H_ replacement, like other forms of receptor editing, occurs primarily in the immature B cell population to rescue non-functional or autoreactive recombinants (Zhang et al., 2004; Lutz et al., 2006), but some studies suggest that V_H_ replacement may be possible in mature B cells (Hikida et al., 1996; Han et al., 1997; Papavasiliou et al., 1997; Hertz et al., 1998; Nussenzweig, 1998). Somewhat paradoxically, V_H_ replacement, which is purported to be a primary mechanism for resolving self-reactive recombinations, can itself result in antibodies with autoreactive characteristics (Klonowski and Monestier, 2000; Zhang et al., 2003). V_H_ replacement was observed first in transformed murine pre-B cells (Kleinfield et al., 1986; Reth et al., 1986), with subsequent studies identifying V_H_ replacement *in vivo* (Taki et al., 1993; Chen et al., 1995). In the most informative work done on V_H_ replacement in the human repertoire, a genetic fingerprint of V_H_ replacement was identified in the human peripheral blood repertoire (Zhang et al., 2003). Identification of V_H_ replacement events in the peripheral repertoire relies on detection of short pentameric sequences that are located between the cRSS and the 3′ end of V genes. These pentamers remain even after V_H _replacement, providing an identifiable remnant of the replaced V gene. Short pentameric sequences are easily mimicked through random N-addition, making reliable detection of V_H_ replacement difficult. Therefore, estimates of V_H_ recombination frequency in the peripheral blood repertoire have varied widely, from 5 to 22% of the total repertoire (Zhang et al., 2003; Koralov et al., 2006; Watson et al., 2006).

## SOMATIC HYPERMUTATION

In humans and in mice, diversification of the secondary antibody repertoire, which arises in response to antigenic stimulus, is accomplished primarily through SHM (Brenner and Milstein, 1966; Kelsoe, 1994). Naïve, antigen-inexperienced B cells undergo the SHM process upon recognition of an infectious agent. It is through the SHM process, which occurs primarily in secondary lymphoid tissue, that hosts mutate the variable region of their antibody genes (MacLennan et al., 1992; Li et al., 2004). Many of these mutations have no effect on antigen recognition and many have deleterious effects on either antigen recognition or proper folding of the antibody protein. Some mutations, however, produce antibodies with improved affinity for the target pathogenic epitope (Casali et al., 2006). Thus, the SHM process provides a basis for the positive selection of high-affinity antibodies that are characteristic of a mature immune response (MacLennan, 1994).

Many components of the SHM machinery are known, but the complete process and the mechanisms by which it is targeted specifically to the immunoglobulin loci are still poorly understood. SHM introduces point mutations at a frequency of approximately 10^-3^ mutations per base pair, which is about 10^6^-fold higher than the rate of spontaneous mutation in other genes (Rajewsky et al., 1987). Mutations begin approximately 150-bp downstream of the transcription start site and the mutation frequency decreases exponentially with increasing distance from the transcription start site (Rada and Milstein, 2001). Activation-induced cytidine deaminase (AID) is required for SHM and initiates the SHM process by the deamination of C nucleotides (Muramatsu et al., 1999, 2000). Deamination results in a U–G mismatch, and several possible processes result in the error-prone repair of the mismatch. Although the precise mechanism(s) responsible for error-prone repair during SHM are not known, several DNA repair mechanisms have been shown to be critical to the SHM process, including base excision repair and mismatch repair (Phung et al., 1998; Rada et al., 1998; Wiesendanger et al., 2000; Di Noia and Neuberger, 2002; Zheng et al., 2005).

## SOMATIC HYPERMUTATION-ASSOCIATED INSERTIONS AND DELETIONS

Although the SHM process typically results in single nucleotide substitutions, deletion of germline nucleic acids or insertion of non-germline nucleic acids does occur in association with SHM (Goossens et al., 1998; Wilson et al., 1998a; Bemark and Neuberger, 2003). These insertions and deletions (indels) are rare, with SHM-associated (SHA) indels estimated to be present in 1.3–6.5% of circulating B cells (Goossens et al., 1998; Wilson et al., 1998a; Bemark and Neuberger, 2003). Short SHA indels are much more common than long SHA indels, with most insertions and deletions being 1–2 codons in length (Goossens et al., 1998; Wilson et al., 1998a; Bemark and Neuberger, 2003). Although infrequent, SHA insertion and deletion events add substantially to the diversity of the human antibody repertoire (Wilson et al., 1998b; de Wildt et al., 1999; Reason and Zhou, 2006).

Somatic hypermutation-associated insertions and deletions also have been shown to play a critical role in the antibody response against viral and bacterial pathogens, including HIV, influenza, and *Streptococcus pneumoniae* (Zhou et al., 2004; Walker et al., 2009, 2011; Wu et al., 2010a; Krause et al., 2011; Pejchal et al., 2011). Of particular interest, structural analysis of an SHA insertion in the anti-influenza antibody 2D1 identified a substantial structural alteration induced by the insertion (Krause et al., 2011). This insertion, although located in a FRs, caused a large conformational change in a CDR and allowed antibody–antigen interactions that were sterically hindered without the insertion-induced conformational change. In addition to 2D1, the extremely broad and potently neutralizing HIV antibody VRC01 contained a six nucleotide deletion in the CDR1 of the light chain (CDR-L1; Wu et al., 2010a). This SHA deletion shortened the CDR-L1 loop, thereby removing potential clashes with loop D of the HIV envelope protein and allowing direct interaction between the HIV antigen and the CDR-L2 loop of VRC01 (Zhou et al., 2010).

## ANTIBODY COMPLEMENTARITY DETERMINING REGIONS

Antibody CDRs (also referred to as hypervariable regions) are the primary region of antigen recognition, contain extensive sequence diversity even among germline genes, and are targeted preferentially for affinity maturation, making them the most variable regions of the antibody gene (Capra and Kehoe, 1975; Kabat et al., 1992). There are several structural and genetic reasons for the preferential targeting of CDRs by SHM. Genetically, SHM is known to preferentially target the WRCY hotspot motif (or its reverse complement, RGYW; Dörner et al., 1998), and the frequency of these hotspots is increased in CDRs (Wagner et al., 1995; Shapiro and Wysocki, 2002; Pham et al., 2003). Further, codon usage is biased in CDRs toward codons that are easily mutable, enhancing the likelihood that a nucleotide substitution induced by SHM results in an amino acid change (Motoyama et al., 1991; Wagner et al., 1995; Kepler, 1997). Structurally, the CDRs are largely loop-based, which make them sufficiently flexible to incorporate the substitutions and short indels introduced by SHM without compromising structural integrity. FRs, by contrast, are highly structured and less able to accommodate somatic mutations (Celada and Seiden, 1996).

## AFFINITY MATURATION AND ANTIGEN CONTACT BY ANTIBODY FRAMEWORK REGIONS

While much affinity maturation is focused on the CDRs, there are other regions that are important to antigen recognition. T cell receptors (TCRs) contain a fourth hypervariable region (HV4, sometimes referred to as CDR4), which is highly variable, surface-exposed, and involved in superantigen and accessory molecule recognition (Choi et al., 1990; Garcia et al., 1996; Li et al., 1998). We have recently used high-throughput sequencing approaches to determine the sequence of thousands of antibody genes containing SHM-associated insertions and deletions (SHA indels), which revealed significant differences between the location of SHA indels and somatic mutations (Briney et al., 2012c). Further, we identified a cluster of insertions and deletions in the antibody FR3 region that corresponds to the HV4 in TCRs.

Emerging evidence suggests that an HV4-like region may exist in antibodies as well as TCRs. Recent crystallographic work on the anti-influenza antibody BR6261 has shown that the HV4-like region of FR3 was somatically mutated (Throsby et al., 2008) and directly contributed to antigen binding (Ekiert et al., 2009). The anti-influenza antibody 2D1 contains a three-codon insertion in a HV4-like region of FR3 which, while not directly involved in antigen recognition, causes a critical conformational shift in nearby CDRs that is required for antigen recognition (Krause et al., 2011). A unique example of HV4-like contribution to antigen recognition is the anti-HIV antibody 21c (Diskin et al., 2010). 21c binds to the HIV co-receptor binding pocket, which is only exposed following binding of CD4, the primary host receptor. Interestingly, while the majority of the binding surface of 21c is in contact with the HIV envelope protein, the HV4-like region of 21c binds to CD4, forming a cross-protein epitope. In addition to 21c, the broadly neutralizing anti-HIV antibody VRC03 contains a surprisingly long seven-codon insertion in the HV4-like region of FR3 (Wu et al., 2010a). Finally, the HV4-like FR3 region of antibody heavy chains of the V_H_3 family has been shown to interact with Staphylococcal protein A, a known superantigen (Potter et al., 1996), mimicking the superantigen-binding activity of the HV4 region in TCRs. While the HV4-like regions that have been identified to date are not somatically mutated to the same extent as antibody CDRs, the ability of this HV4-like region to tolerate a substantial number of somatic mutations and genetic insertions suggests the existence of a somewhat flexible region that has an under-appreciated ability to accommodate affinity maturation modifications.

## CONCLUSION

V(DD)J recombination, SHA indels, and antigen contact by non-CDR antibody regions, while secondary to V(D)J recombination and SHM as mechanisms of antibody diversification, contribute substantially to antibody diversity. Each of these secondary affinity maturation mechanisms allows for the generation of unique genetic or structural elements that have been shown to be important to the humoral response against a variety of viral and bacterial pathogens including HIV, influenza virus, staphylococci and pneumococci. These secondary affinity maturation events are much less common than SHM and, as a consequence, are more difficult to study effectively. The advent of next-generation sequencing technology has made it is possible to obtain thousands or millions, and soon to be billions, of antibody sequences (Boyd et al., 2009, 2010; Wu et al., 2010b; Prabakaran et al., 2011; Briney et al., 2012d). It is likely that over the coming years, this digital flood of antibody sequence data will allow a much more complete understanding of these secondary affinity maturation events. For example, current technologies for isolating antigen-specific antibodies from human blood or bone marrow cells are relatively inefficient and result in stochastic discovery of unique antibodies. High-throughput sequence analysis techniques now allow comprehensive definition of all expressed antibody sequences in samples, even to the scale of analyzing all antibody sequences in leukopacks containing most of the circulating B cells in an individual at a time point. Novel methods under current development for determining phylogenetic relationships among expressed antibody sequences may allow us to define the path of somatic mutation from unmutated ancestor sequences to the final affinity-matured antigen-specific sequence. Likely, these studies will reveal that B cell clones that develop following antigen stimulation do not follow linear paths of development, but rather diverge into complex families with multiply branched phylogenies. Such studies should greatly broaden our understanding of the molecular and genetic events occurring in the B cell repertoire following antigen stimulation.

## Conflict of Interest Statement

The authors declare that the research was conducted in the absence of any commercial or financial relationships that could be construed as a potential conflict of interest.
